# Soft Urinary Bladder Phantom for Endoscopic Training

**DOI:** 10.1007/s10439-021-02793-0

**Published:** 2021-05-17

**Authors:** Eunjin Choi, Frank Waldbillig, Moonkwang Jeong, Dandan Li, Rahul Goyal, Patricia Weber, Arkadiusz Miernik, Britta Grüne, Simon Hein, Rodrigo Suarez-Ibarrola, Maximilian Christian Kriegmair, Tian Qiu

**Affiliations:** 1 Cyber Valley Research Group, Institute of Physical Chemistry, University of Stuttgart, Pfaffenwaldring 55, 70569 Stuttgart, Germany; 2 Micro Nano and Molecular Systems Lab, Max Planck Institute for Intelligent Systems, Heisenbergstr. 3, 70569 Stuttgart, Germany; 3 Department of Urology & Urosurgery, University Medical Centre Mannheim, Faculty of Medicine, University of Heidelberg, Theodor-Kutzer-Ufer 1-3, 68167 Mannheim, Germany; 4 RaVeNNA 4pi – Consortium of the German Federal Ministry of Education and Research (BMBF), Mannheim, Germany; 5 Department of Urology, Faculty of Medicine, University of Freiburg - Medical Centre, Hugstetterstr. 55, 79106 Freiburg, Germany

**Keywords:** Organ phantom, Cystoscopy, Endourology, Tumor biopsy, Medical education, Surgical simulation

## Abstract

Bladder cancer (BC) is the main disease in the urinary tract with a high recurrence rate and it is diagnosed by cystoscopy (CY). To train the CY procedures, a realistic bladder phantom with correct anatomy and physiological properties is highly required. Here, we report a soft bladder phantom (FlexBlad) that mimics many important features of a human bladder. Under filling, it shows a large volume expansion of more than 300% with a tunable compliance in the range of 12.2 ± 2.8 – 32.7 ± 5.4 mL cmH_2_O^−1^ by engineering the thickness of the bladder wall. By 3D printing and multi-step molding, detailed anatomical structures are represented on the inner bladder wall, including sub-millimeter blood vessels and reconfigurable bladder tumors. Endoscopic inspection and tumor biopsy were successfully performed. A multi-center study was carried out, where two groups of urologists with different experience levels executed consecutive CYs in the phantom and filled in questionnaires. The learning curves reveal that the FlexBlad has a positive effect in the endourological training across different skill levels. The statistical results validate the usability of the phantom as a valuable educational tool, and the dynamic feature expands its use as a versatile endoscopic training platform.

**Electronic supplementary material:**

The online version of this article (10.1007/s10439-021-02793-0) contains supplementary material, which is available to authorized users.

## Introduction

Bladder cancer (BC) is the second most frequent cancer in the urinary tract.^13^ Cystoscopy (CY) is a basic and frequently applied endoscopic procedure to examine the urinary bladder and its possible pathological alterations,^8^ including BC. BC is characterized by a high recurrence rate of up to 70% and progression.^24^ It is caused by many reasons and one of them is missing of the lesions due to insufficient endoscopic exploration.^21^ The bladder does not have a simple spherical shape and is a highly-compliant organ that can expand for several times in volume, presenting diverse anatomic variations.^4^ To examine the entire bladder surface and thereby assess the potential lesions require endoscopic skills of urologists. Previous studies have shown that less experienced surgeons subjectively visualize a lower proportion of the bladder surface 27 and that the first 45 procedures of the transurethral resection of bladder tumors (TURBT) performed are associated with higher BC recurrence rates,^22^ which demonstrate the essential needs for cystoscopic training.

A growing surgical consensus promotes the requirements for training of surgical procedures on organ phantom before their exploration in human.^23^ Lurie *et al*. reported a distendable bladder phantom for white light cystoscopy.^17^ Blankstein *et al*., reported a 3D printed urinary model for ureteroscopy and validated the model for training.^6^ Jaksa *et al*. reported a laparoscopic trainer with multiple organ models for the radical prostatectomy.^14^ Commercial simulators were also used in medical studies, for example by de Vries *et al*. for urological residency training.^11^ However, the existing bladder phantom fail to mimic the physiological tissue properties and important bladder anatomical structures, which are very important to provide realistic feedbacks to the surgeons during the surgical simulations and it directly affects the training outcome. Recently, we reported soft organ phantom that allow realistic surgical procedures and the quantitative evaluation of the surgical outcome, for example, endoscopic procedures in soft kidney phantom with the detailed collecting system^2^ and electrocautery surgeries in benign prostate hyperplasia (BPH) phantom^9^,^15^ and the needle insertion for biopsy in liver phantom.^26^ To the best of our knowledge, currently there is no soft bladder phantom available that represents realistic compliance as well as the detailed anatomical structures in the bladder for endoscopic training.

In this paper, we report soft bladder phantom that exhibits realistic physiological compliance and detailed vasculature on the inner wall, named FlexBlad. By combining high-resolution 3D printing and polymer injection molding techniques, FlexBlad possesses realistic anatomic structures such as vasculature and bladder tumors. The particular design of its compliance provides both high-fidelity visual and haptic feedback during the training as in a real intervention. The FlexBlad can serve as a realistic platform for the medical training of endoscopic procedures, such as white light cystoscopy (WLC), as well as for the testing of new medical devices and techniques.

## Materials and Methods

### Design of the 3D Model for FlexBlad

**Figure 1:**
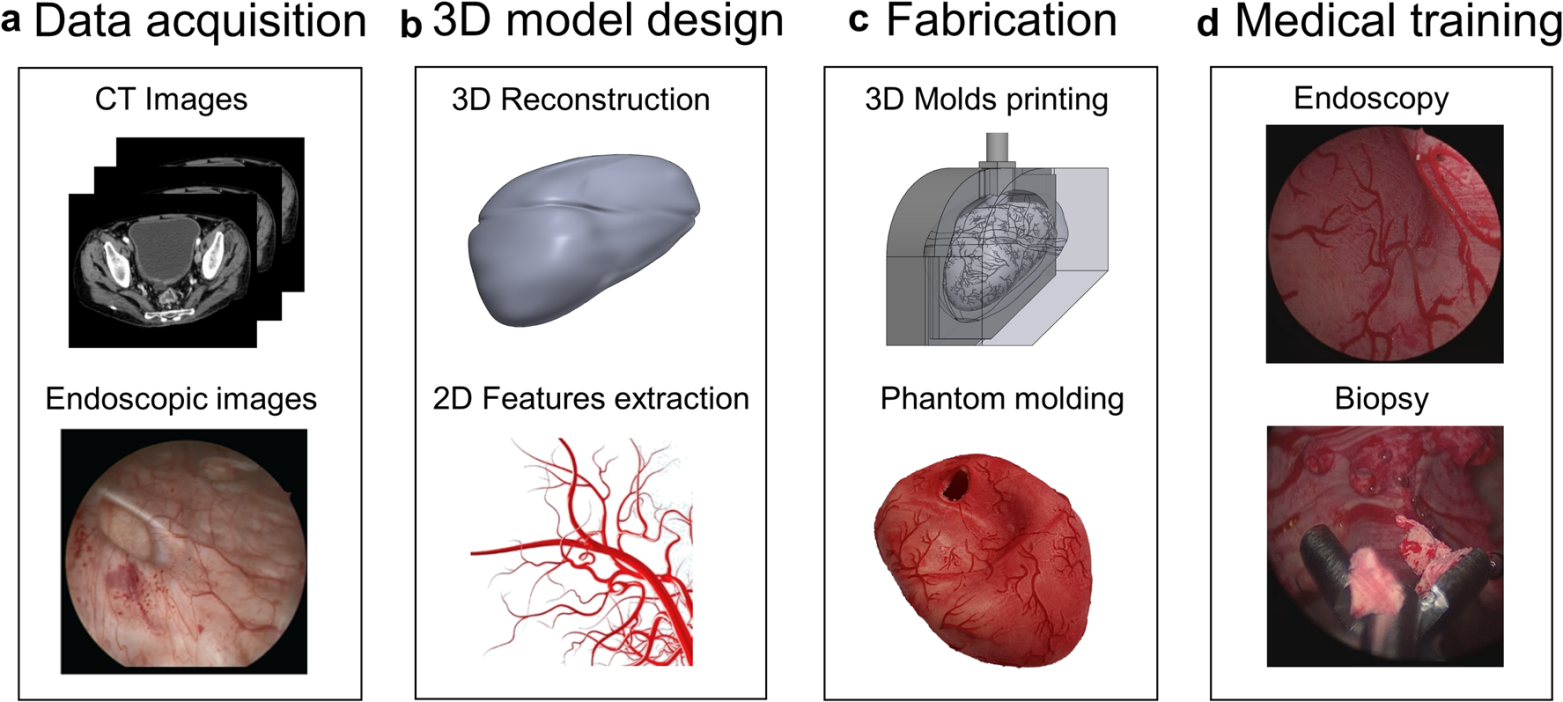
Schematic of the workflow to develop the soft bladder phantom for the endoscopic training.

**Figure 2:**
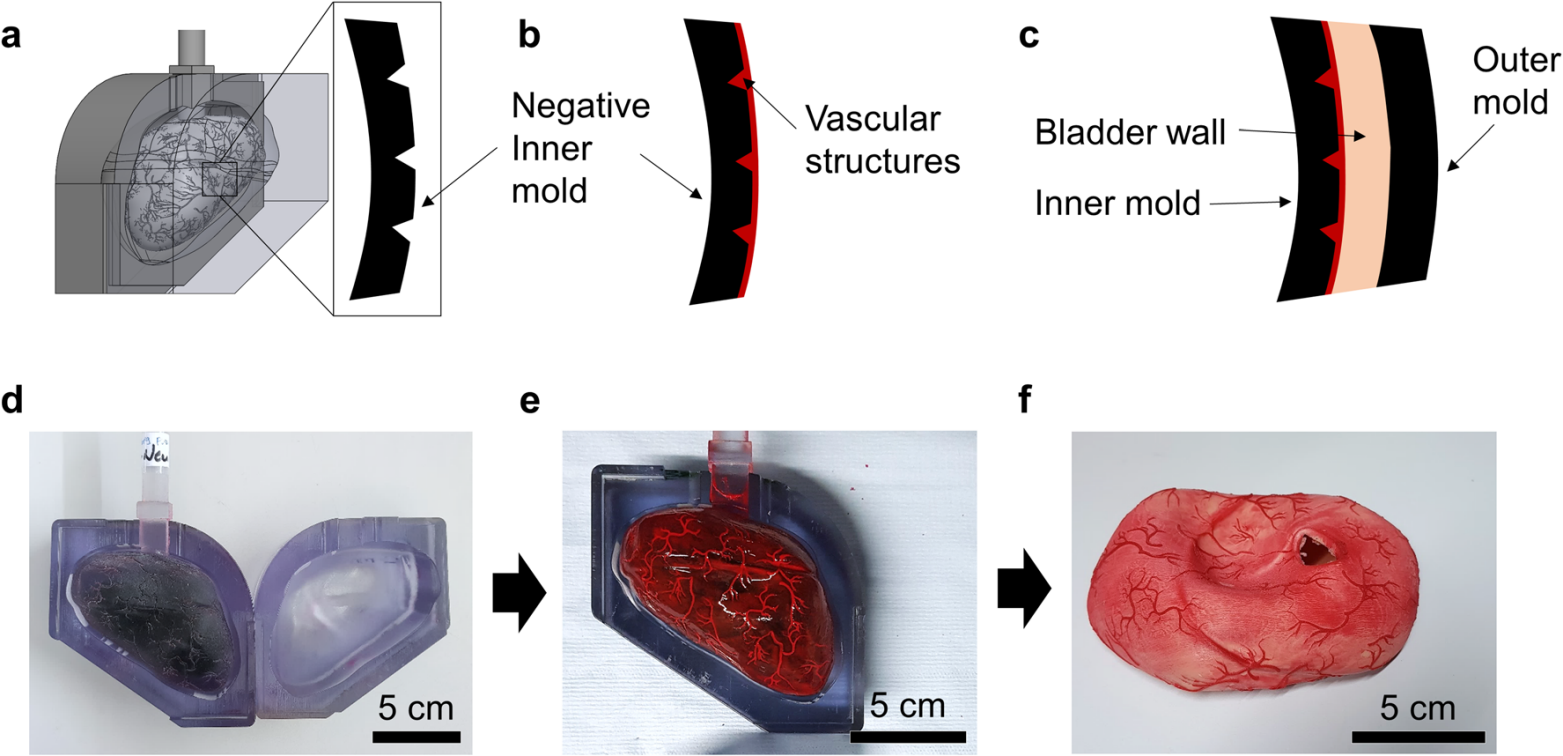
Fabrication process of the bladder phantom. (a–c) schematics of the cross-sectional view of the two-step molding process, and (d–f) photos of the corresponding steps.

CT (computer tomography) images of ten human patients (44–72 y) with filled bladders were obtained from the University Medical Centre Mannheim, approved by the Ethical Committee II, University of Heidelberg, Germany, under the reference number: 2015-549 N-MA. As illustrated in Fig. 1, the CT images were extracted with an open-source medical image viewer (Horos™, horosproject.org, Purview, Annapolis, MD, USA), reconstructed, and exported as.stl files. The most representative bladder form was selected according to the medical experts and the model was scaled to 150 mL in volume at a relaxed state. Anatomical details, including the urethra, the interureteric bar, and the blood vessels, were designed based on cystoscopic videos in consultation with medical experts and added onto the surface of the model, using computer-aided design (CAD) software (Solidworks 2019, Dassault Systèmes Corp., Waltham, MA, USA). Three molds were designed: (1) an inner mold that matches the relaxed volume and the shape of the bladder and includes important anatomical structures, such as interureteric bar, and the vascular network; (2) an outer mold to define the thickness of the bladder wall; and (3) a urethra mold to define the shape and thickness of the urethra. In total, three different negative bladder molds (inner mold, an outer mold, and a urethra mold) were designed, as shown in Fig. 2. FlexBlad’s wall was prepared by a molding process. The outer molds being prepared in three different wall thicknesses, *i.e.,* 2 mm, 3 mm, and 5 mm, allowed FlexBlad to have respective compliance.

### Fabrication of FlexBlad

The negative molds were printed with VeroClear^®^ material in a 3D printer (Object 260 Connex, Stratasys, Israel). In order to realize its high flexibility, a soft silicone material (Ecoflex 00-20, Smooth-on, Macungie, PA, USA) was chosen to fabricate the phantom. First, a mold release agent was evenly sprayed on all surfaces of the molds and dried in a fume hood for 30 min. The silicone material was prepared by thoroughly mixing Part A and B in 1:1 of the weight ratio. For coloring, the mixture, 1 wt% of coloring agent (red color for the blood vessels and skin color for the bladder wall and the urethra) was added. The mixture was vigorously stirred mechanically and degassed in a vacuum chamber for 10 min to remove the air bubbles. As shown in Fig. 2, a negative pattern of the blood vessel network on the inner mold was filled with red-colored silicone material, and the viscous material was held in place due to the surface tension in the negative pattern, and the material was solidified using a heat gun at 150 °C. Subsequently, the blood vessel mold was assembled with the outer mold, and a rectangular junction on the inner mold fits with a slot on the outer mold (Fig. 2d) to ensure a precise assembly without any rotation or translation. The same silicone material (skin-colored) was filled between the molds and cured at 65 °C, and the two layers of silicone material firmly adheres to each other. The bladder phantom was released from both molds mechanically. The urethra that was prepared by similar molding steps was assembled to the bladder neck by applying the same silicone material used for the bladder wall and then the silicone material was thermally cured with a heat gun. The tumors (~ 350 – 700 mm^3^ in volume) were fabricated using the same material in different colors, embedded with a magnet (NdFeB, 4 × 4 × 4 mm^3^, Supermagnete, Germany) to allow the repositioning with the control of an external magnet. Biopsy simulation was performed using biopsy forceps (5 Fr., 73 cm, KARL STORZ GmbH, Tuttlingen, Germany).

### Volume Expansion and Compliance Measurement

**Figure 3:**
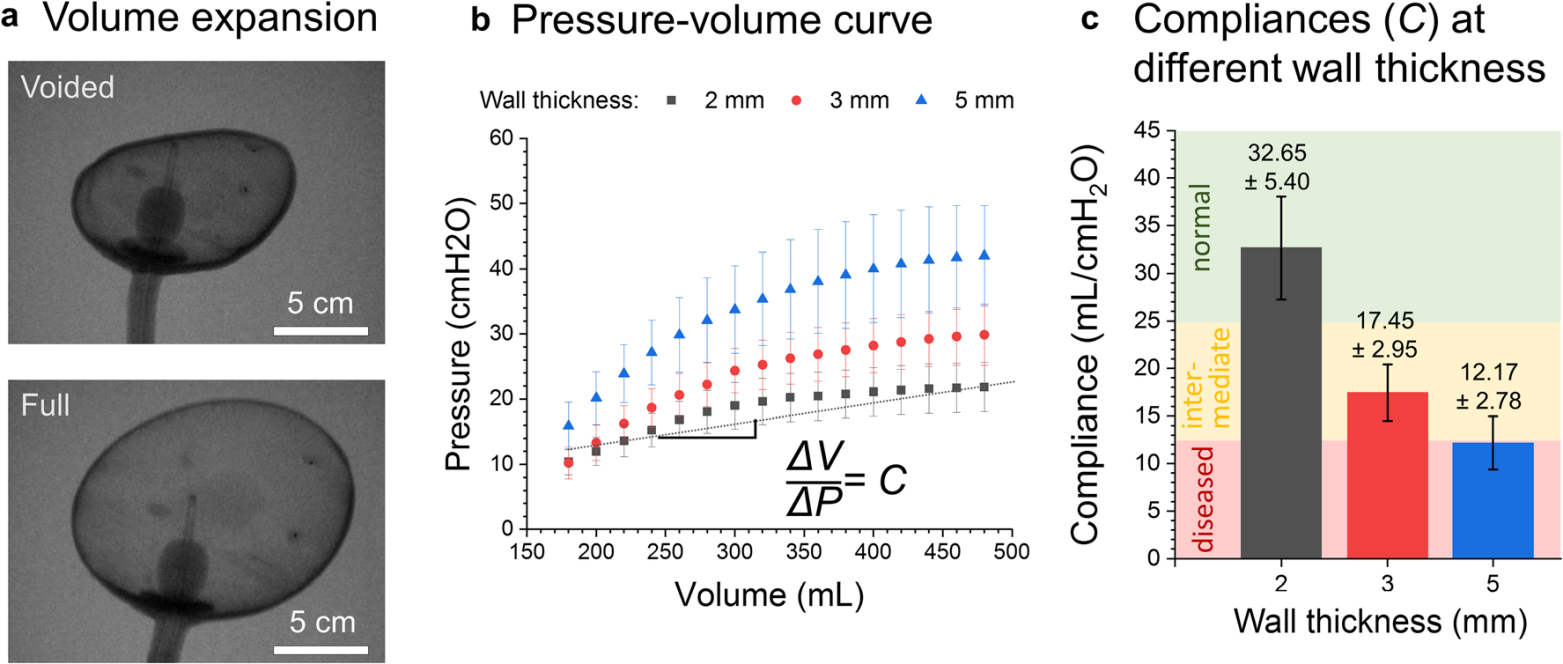
Mimicking the compliance of real human bladders. (a) large volume expansion imaged using X-ray, (b) the pressure–volume curve at different wall thickness of the phantom, and (c) the compliance of the bladder phantom covers different states of the bladder.

The expansion of FlexBlad was examined by X-ray imaging (Uroskop Omnia, Siemens, Germany). For imaging, the contrast agent was filled in by a Foley catheter (16 Fr., 10 mL balloon inflation, Uromed, Oststeinbek, Germany). The compliance of the bladder phantom was measured using an electronic pressure sensor (SMI-1A, IntraSense™, Silicon Microstructures Inc., Milpitas, CA, USA). The sensor was mounted on a rigid stick and fixed to the bottom of the bladder phantom. Water was filled in through the soft urethra with a syringe until a total volume of 480 mL. The urethra opening was sealed with a rubber band to ensure no water leakage. The volume-pressure curve of bladder phantom with three different wall thicknesses of 2, 3, and 5 mm were measured for five times independently on each phantom, respectively. The results were averaged and plotted in Fig. 3.

### Endoscopic Evaluation

When simulating a cystoscopy examination, the bladder phantom was placed in a box, which was covered with a sheet of silicone material to mimic the abdominal skin. Surgeons can press on the outer surface of bladder phantom *via* the “skin” to feel and deform the bladder. Endoscopic evaluation was performed using a flexible cystoscope (15 Fr. flexible cystoscope deflected up to 210° and down to 140°, KARL STORZ GmbH, Tuttlingen, Germany) to observe and validate the FlexBlad. Three important features: blood vessel, dome deformation, and bladder tumor, were compared with a real human bladder. For validation, content validity (CtV), face validity (FV), and construct validity (CsV) were performed^20^ in two urological centers (University Medical Centre Mannheim and University Medical Centre Freiburg) by twenty surgeons in total. After a standardized cystoscopic exploration with the flexible cystoscope, the surgeons were asked to fill in a Likert-scale-based questionnaire about seven aspects (size/anatomy, bladder tissue, urethra, interuretheric bar, haptic feedback, training tool, and recommendation to students) from 1 (strong disagreement) to 5 (strong agreement) for CtV. An additional questionnaire allowed to score the usability of FlexBlad’s system according to system usability scale (SUS) for FV. The SUS score system is an established tool to evaluate the usability of systems.^5^,^7^ Like a Likert scale, the user rates ten factors: five positive aspects (Frequent usage, Functions well integrated, Easy to use, Usage quickly learnable, and Usage confident) and five negative aspects (Too much inconsistency, Learn a lot before use, Technical support needed, Unnecessary complex, and Cumbersome use). CsV measured the ability of a simulator to differentiate between different skill levels.^3^ This was determined using the time records of FlexBlad CY from two different expert groups of surgeons (who has performed less or more than 200 CY), each in four repetitive training sessions. For statistics, the Mann–Whitney *U* test compared the intergroup performance using Origin (2018, OriginLab, Northampton, MA, USA).

## Results

### Anatomic Properties of FlexBlad

**Figure 4:**
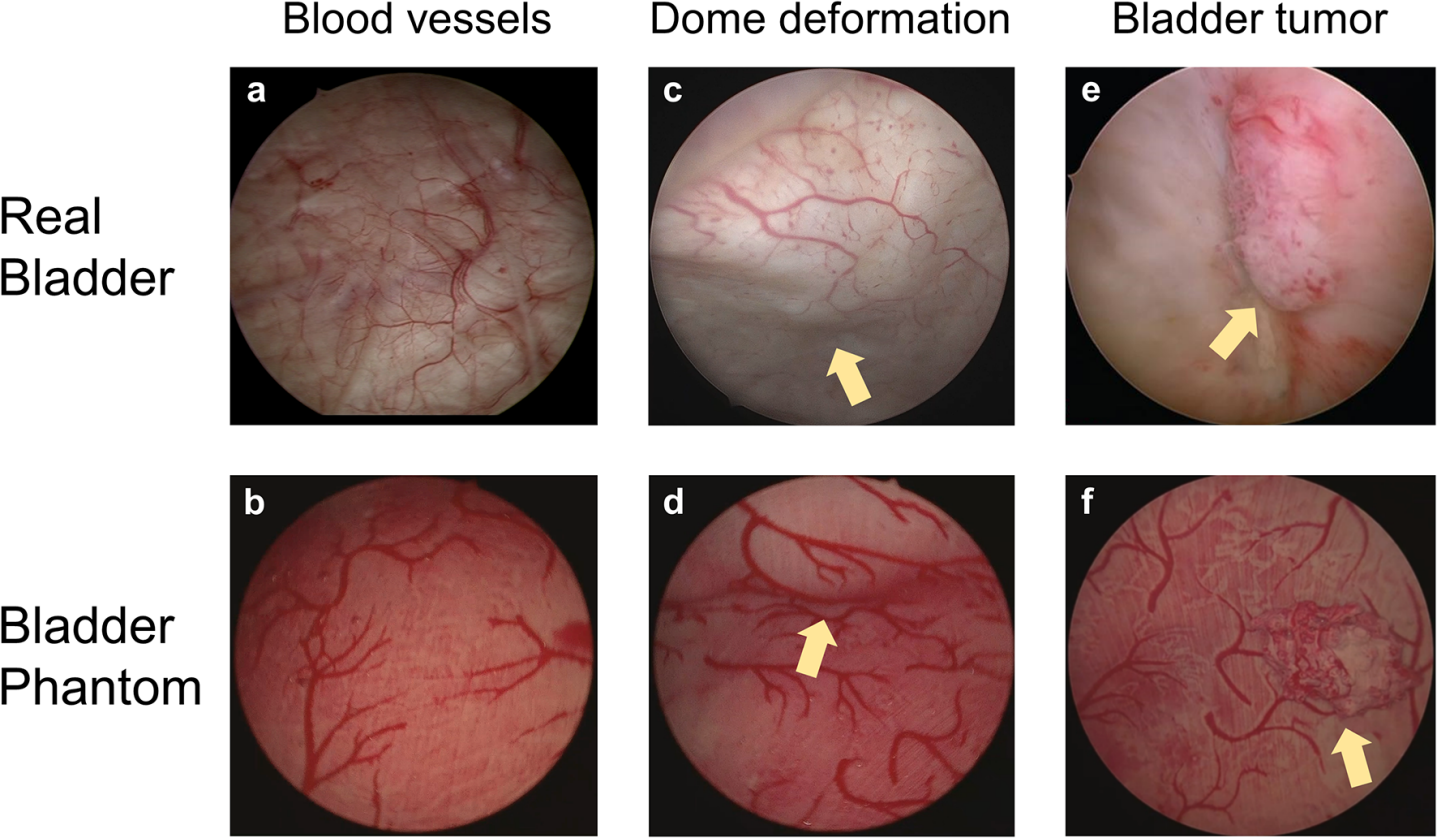
Comparison of the endoscopic view of the real bladder (upper panel) and the bladder phantom (lower panel). The arrows indicate the position of the deformed bladder dorm in (c) and (d), and the tumors in (e) and (f).

The bladder phantom with detailed vascular structures were realized by the multiple molding process on 3D-printed molds (Fig. 2). 3D printing offered a spatial resolution to achieve sub-millimeter complicated structures that are designed in the digital bladder model. The designed blood vessel network was engraved on the outer surface of the inner mold (Fig. 2a). By filling viscous silicone material in the narrow negative-patterned network, the material remained in the gap until curing. Then, the outer mold was assembled and covered the inner mold to unify to the phantom bladder wall. The step-wise assembled parts were well-integrated and no delamination was observed under large strain (> 200%). This fabrication method replicates the vascular network down to 0.5 mm width with complicated 3D geometries, mimicking the human bladder vascular network. Various endoscopic explorations of the phantom led to a continuous improvement of the artificial bladder tissue towards the final results (Fig. 4, Supplementary Video S1).

### Bladder Wall and the Compliance

Since the FlexBlad was made of silicone materials, it showed large volume expansion, as confirmed by X-ray imaging (Fig. 3a). During this exploration FlexBlad voiding under X-ray came very close to a realistic voiding cystourethrography. The expansion of the phantom was repeatedly carried out with no damage observed. The compliance of FlexBlad, defined as the volume expansion over the internal pressure increase in the unit of mL cmH_2_O^−1^,^1^ was measured in a range from 12.2 ± 2.8 – 32.7 ± 5.4 mL cmH_2_O^−1^ (Fig. 3c). This range covered physiological and pathological bladder compliance values. Although the values vary depending on measurement conditions and methods, physiological human bladder compliance is considered to be > 25 mL cmH_2_O^−1^.^29^ Compliance ratings from 12.5 – 25.0 mL cmH_2_O^−1^ are considered as intermediate bladder compliance, values below 12.5 mL cmH_2_O^−1^ are defined as poor compliance.^28^

### Comparison to a Real Bladder

Figure 4 compares endoscopic images of the FlexBlad exploration to human cystoscopic images focusing on the aspects of vascular network depiction, bladder dome deformation and bladder tumors. As shown in Figs. 4a and 4b, the fine and arbitrary shape of the blood vessel was well replicated in the phantom. The vascular network allowed surgeons to visually track structures and position while operating the endoscope. As a result, hand–eye coordination could be strengthened. In addition, the vascular network enables training in the systematic examination of the urinary bladder by recognizing structures but also facilitates orientation.

In Figs. 4c and 4d, the suprapubic pressure to allow the visualization of the anterior bladder wall was observed comparing to human cystoscopic examinations. The haptic feedback was verified and lead to high satisfaction (see below), which attested FlexBlad’s abilities for anterior wall visualization to be close to reality.

**Figure 5:**
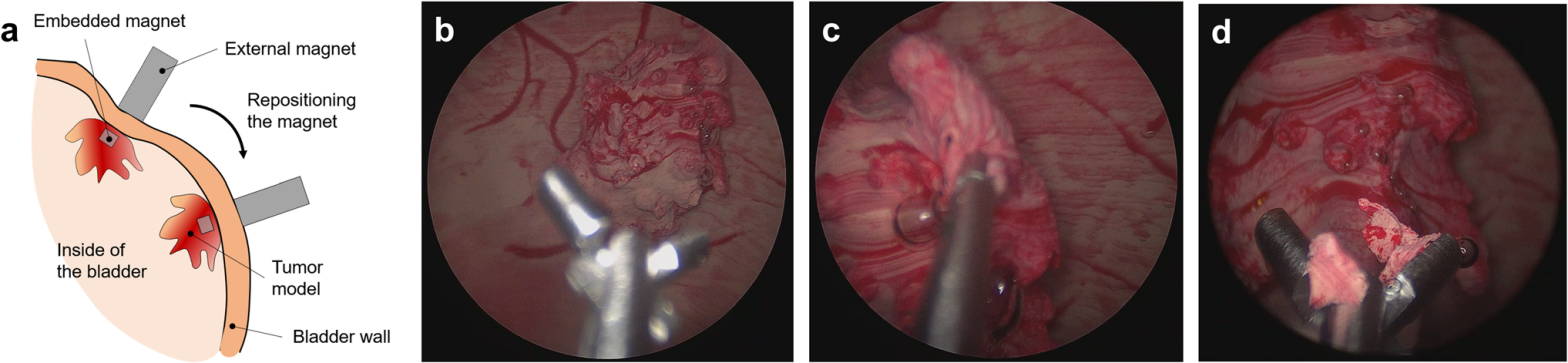
Reconfigurable bladder tumor in the phantom for endoscopic training. (a) Schematic illustration of changing the tumor’s position inside the phantom using an external magnet; (b–d) Sequential snapshots of the biopsy simulation of a papillary tumor on the bladder wall using forceps through the tool channel of an endoscope.

Figures 4e and 4f compare the real and the sham bladder tumors. The sham tumor was fixed by two attracting magnets (one embedded in the tumor and the other out of the bladder) so that they retained their position but the position was also reconfigurable. As shown in Fig. 4f, a 2 cm-diameter ellipsoidal papillary tumor is attached to the inner bladder wall and can be clearly visualized under the endoscopic view (see also Supplementary Video S2). Their positioning at certain locations that were difficult to inspect allowed an objective control of the complete bladder visibility (Fig. 5). Additionally, the repositioning offers the chance to increase the diversity and the difficulty in endoscopic training. Furthermore, repeated tumor biopsies could be performed under real conditions (Figs. 5b–5d, see also Supplementary Video S3).

### Endoscopic validation of the FlexBlad

**Figure 6:**
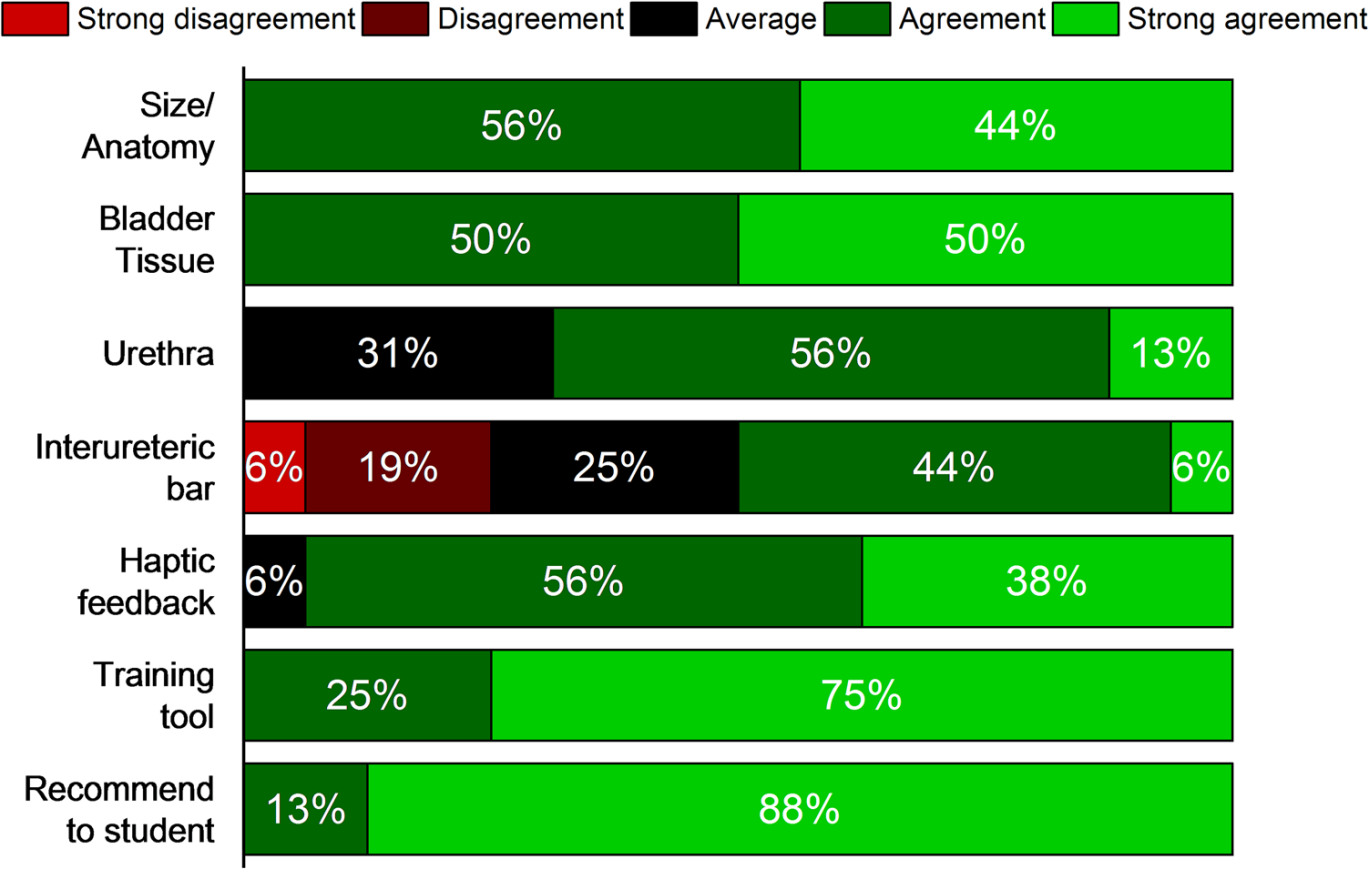
Survey results of the bladder phantom for endoscopy using a 5-point Likert scale on seven aspects to verify the realization of important features on the phantoms to be used as a medical tool (*n* = 16). The results indicate that most subjects agree that the FlexBlad is a valuable surgical training tool.

**Figure 7:**
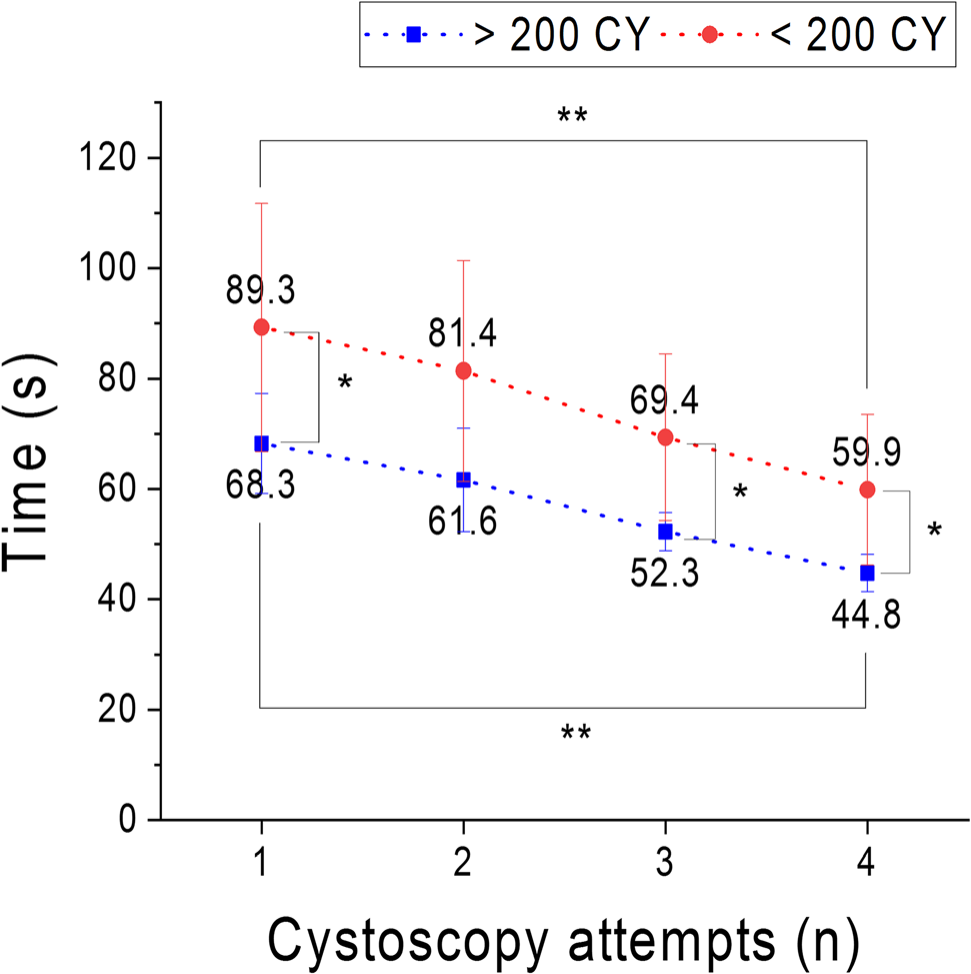
Comparison of the execution time for cystoscopy simulation by two groups of different skilled surgeons for construct validity (CsV) over four times attempts (*n* = 16), ** *p *< 0.01, * *p *< 0.05.

FV was assessed by analyzing the responses of SUS questionnaires (*n* = 20) and translated into a SUS score of 81.9 ± 5.7. The score was contributed by a high rate in positive aspects (Frequent usage: 4.3 ± 0.7; Functions well integrated: 3.4 ± 0.7; Easy to use: 4.2 ± 0.7; Usage quickly learnable: 4.4 ± 0.6; and Usage confident: 4.7 ± 0.5) and low rate in negative aspects (Too much inconsistency: 1.7 ± 0.5; Learn a lot before use: 1.5 ± 0.5; Technical support needed: 1.6 ± 0.6; Unnecessary complex: 1.8 ± 0.4; and Cumbersome use: 1.7 ± 0.5). This rate proves that the FlexBlad has an ‘excellent’ level of usability. CtV was assessed by 5-point Likert-scale questionnaires to urologists (*n* = 16). The highest approval ratings were obtained for the attestation that FlexBlad is a useful phantom (4.8 ± 0.5), which should be recommended to medical students (4.9 ± 0.3). Further approving results were achieved for size and anatomy (4.4 ± 0.5), realistic bladder tissue (4.5 ± 0.5), and realistic haptic feedback (4.3 ± 0.6). Intermediate scores were obtained for the structures of the urethra (3.8 ± 0.7) and the interureteric bar (3.3 ± 1.1). The distribution of the data is also presented on the percentile scale in Fig. 6. In CsV evaluation, as shown in Fig. 7, the execution time of the CY significantly decreased from the first to the fourth repetitions in both groups of the experienced urologists (68.3 ± 9.1 s versus 44.8 ± 3.4 s, *p *= 0.0002) and the less experienced urologists (89.3 ± 22.5 s versus 59.9 ± 13.7 s, *p *= 0.007). (Figure 7) Intergroup comparison showed that experienced urologists were significantly faster and the performance was more stable in all attempts than the less experienced ones (1^st^: *p *= 0.04; 2^nd^: *p *= 0.05; 3^rd^: *p *= 0.005; 4^th^: *p *= 0.01).

## Discussion

The urinary bladder has individual characteristics in shape and volume confronting CY beginners with interventional challenges that need to be trained and mastered *ex vivo*. In order to train CY in a realistic environment, bladder phantom are required that mimic the physical properties of a human bladder as accurately as possible.

The technical evaluation shows that the FlexBlad exhibits a realistic compliance covering normal (> 25 mL cmH_2_O^−1^) and diseased bladders (< 12.5 mL cmH_2_O^−1^). Although the Ecoflex material used in this study (with an elastic modulus ~ 55 kPa^12^,^16^) is softer than the bladder tissue (more than 250 kPa^10^,^19^), we are able to match the overall compliance of the bladder and also tune it in a large range by engineering the wall thickness of the phantom. The low bladder compliance, which can be caused by several reasons, including radiation therapy, is associated with reduced bladder capacity^18^,^25^ and results in difficult endoscopic conditions. Training on FlexBlad in advance can give a sense of how to handle the difficulties in various situations. The ability of adjustable compliance is not only used for training purposes but also to test surgical instruments such as catheters and pressure sensors.

The 3D arbitrary design of complex structures and fabrication of bladder phantom grant important features, for example, large expansion and optical resemblances: blood vessel network, dome deformation, and tumor integration under endoscopic view. The detailed structures create a realistic environment to train and simulate surgical procedures with realistic haptic feedback. The current bladder phantom has a uniform wall thickness at different locations, which can be further improved in the future. For example, it will be interesting to image the wall thickness distribution in real bladders and mimic it in a phantom to study the impact of wall thickness on the deformation of the organ. The bladder model will be of interests for the training of many endoscopic and laparoscopic procedures in urology, for example, a transurethral resection of bladder tumors (TURBT) and an urethrovesical anastomosis (UVA) after the radical prostatectomy.

Commercial and previously reported organ phantom are usually static and often present fixed anatomic structures or lesions that cannot be changed in positions. This leads to habituation of the anatomy and thus presumably a reduced training effect in repeated training sessions. To correct the flaw, FlexBlad possesses the ability to reconfigure the tumor position by quickly relocating it using an external magnet. The method is simple and robust. The anchored tumor model is held at the desired position with the magnetic force that is strong enough for a biopsy procedure. The possibility of a simulated application of suprapubic pressure provides an additional realistic feature of cystoscopy. These embedded dynamic features of the phantom, together with the ability to vary in volume, create divergent intervention conditions, which potentially enhance the training effect.

The remarkable features and convenience of the FlexBlad for cystoscopy training are verified with excellent values in the subjective validations (FV and CtV), which are represented by the Likert-scale based evaluation and the SUS-score. To see a training effect on FlexBlad, the time sequences of the training sessions of urologists with different levels were compared. The result of the training (CsV) verifies both an improvement in the intervention time over the four sessions in the different expertise groups as well as significant time differences in the group comparison. The multi-centric evaluation indicates that the FlexBlad has a positive effect towards surgical excellence in the endourological training across different skill levels.

In conclusion, we developed a soft bladder phantom, FlexBlad, with complex anatomic structures and matching mechanical properties. The excellent performance was validated with excellent results in CtV, FV, and CsV. Together with the dynamic feature of reconfigurable tumors, the Flexblad enriches the experience and extends the scope of applications as a training tool in urology education and a testing platform for medical instrument.

## Supplementary Information

Below is the link to the electronic supplementary material.

**Video S1** Endoscopic examination of the FlexBlad using a flexible endoscope. Detailed anatomical structures, including vascular network and interureteric plica, can be seen on the inner bladder wall, and the dome of the bladder was deformed by the surgeon for examination. Supplementary material 1 (MP4 25234 kb)

**Video S2** I Endoscopic examination of a tumor model inside the bladder phantom that is fixed to a desired position. Supplementary material 2 (MP4 12795 kb)

**Video S3** Simulation of the biopsy of the tumor model using a pair of forceps through the tool channel of the endoscope. Supplementary material 3 (MP4 7183 kb)
